# Retrograde transduction of dopaminergic cells in substantia nigra of the rhesus monkey

**DOI:** 10.1038/s41598-026-55097-5

**Published:** 2026-06-16

**Authors:** Anya S. Plotnikova, Walter Lerchner, Alexander C. Cummins, Gang Chen, Leonardo Salhani, Vincent D. Costa, Bruno B. Averbeck, Barry J. Richmond, Zayd M. Khaliq, Mark A. G. Eldridge

**Affiliations:** 1 Laboratory of Neuropsychology, National Institute of Mental Health, National Institutes of Health, Bethesda, MD 20892 USA; 2 Nash Family Department of Neuroscience and Friedman Brain Institute, Icahn School of Medicine at Mount Sinai, One Gustave L. Levy Place, New York, NY 10029 USA; 3 Lipschultz Center for Cognitive Neuroscience, Icahn School of Medicine at Mount Sinai, New York, NY 10029 USA; 4 Scientific and Statistical Computing Core, National Institute of Mental Health, National Institutes of Health, Bethesda, MD 20892 USA; 5 Division of Neuroscience, Oregon National Primate Research Center, Beaverton, OR USA; 6 Department of Psychiatry and Behavioral Sciences, Emory University, Atlanta, GA USA; 7 Division of Developmental and Cognitive Neuroscience, Emory National Primate Research Center, Atlanta, GA USA; 8 Cellular Neurophysiology Section, National Institute of Neurological Disorders and Stroke, National Institutes of Health, Bethesda, MD 20892 USA; 9 Aligning Science Across Parkinson’s (ASAP) Collaborative Research Network, Chevy Chase, MD 20815 USA; 10 Biosciences Institute, Newcastle University, Newcastle Upon Tyne, NE1 7RU UK

**Keywords:** Rhesus macaque, Substantia nigra, Dopamine cells, Striatum, Lentivirus, Transduction, Retrograde, Biological techniques, Biotechnology, Neuroscience

## Abstract

Recent advances in molecular tools have changed how researchers approach selective neural modulation, especially in rodent models where germline modifications and viral vector delivery are readily optimized. However, these tools have not advanced as rapidly for nonhuman primates, despite critical need for translational gene therapy models. A key barrier is targeting specific neuronal populations in the larger primate brain with cell-type and circuit specificity. Dopaminergic neurons pose a particular challenge due to their inaccessible ventral midbrain location, where local injection non-selectively targets all dopaminergic neurons. This work provides NHP researchers with a comparison of retrograde viral vectors, highlighting one which achieves efficient dopaminergic neuron transduction, establishing a foundation for combining vectors with cell-type-specific enhancers for basic and translational applications.

**Supplementary Information:**

The online version contains supplementary material available at https://doi.org/10.1038/s41598-026-55097-5.

## Introduction

Dopamine signaling dysfunction is implicated in many psychiatric and neurodegenerative conditions^1^ including substance use disorder^2^, Parkinson’s disease^3–5^, and dystonia^6,7^. The substantia nigra (SN) is the primary source of dopaminergic (DA) innervation in the mammalian central nervous system (CNS)^8,9^. The SN is anatomically and functionally divided into two distinct regions: the substantia nigra pars compacta (SNc), which contains the majority of DA neurons projecting to the striatum, and the substantia nigra pars reticulata (SNr), which consists primarily of GABAergic neurons^10^. Nigrostriatal DA neurons exhibit functional diversity which underlies their essential roles in motor control and goal-directed behaviors^11,12^. As a critical component of the reward circuit, this population of DA neurons contributes to motivation, aversion, and learning processes^13^. Selective modulation of specific sub-populations of DA cells via chemogenetic or other gene therapy methods holds great potential for clinical applications and for basic research on the neural circuitry governing DA-dependent processes^14^. Engineered viral vectors have been used to target the neuromodulatory systems of the nonhuman primate brain^15–19^. Targeting the somata of dopaminergic neurons, however, poses a challenge due to their relatively inaccessible location in the ventral midbrain. Consequently, local injection results in non-selective targeting of all DA neurons within the region, thus requiring a 2-vector system for pathway-specific manipulations^20–22^. An alternative approach is to inject a larger, more accessible brain region, such as the striatum, to retrogradely infect dopamine terminals. Retrograde targeting would allow for selective manipulation of specific dopaminergic pathways while preserving the functionality of other DA projections^23^. In conjunction with retrograde targeting, cell-type-specific regulatory elements, and vectors with selective tropism can constrain expression to DA neurons of interest^24–26^.

Engineering vector tropism allows for high efficacy and specificity in the transduction of target cells^27,28^. Viruses that have been engineered to confer enhanced retrograde transduction properties include a variant of AAV2, AAV2.retro^29^, and HIV-1-based lentiviral vectors^30^ pseudotyped with rabies Fusion Glycoproteins B, C, and E (FuG-B2, FuG-C, and FuG-E, respectively)^31,32^. These engineered viral vectors are endowed with the ability to be internalized by most axon terminals and transported to neuronal cell bodies. HIV-1-based retrograde lentiviral vector, FuG-B2 (‘HiRet’), transduces all brain cell types (glia and neurons), whereas FuG-C (‘NeuRet’) and FuG-E are neuron-specific^32,33^. The rapid propagation and toxicity of conventional retrograde viral techniques, such as rabies and herpes simplex, make pseudotyped lentivirus and AAV more appealing vectors for long-term studies given their lower cytotoxicity and inflammatory potential^33–36^. Efficiency of retrograde transduction is dependent on vector, species, and brain region, among other factors. The AAV2.retro vector, though successful in transducing neurons in cortical and some subcortical structures of the mouse^37^, has shown inconsistencies in transduction efficacy of dopaminergic cells in the SN of both mouse^29^ and NHP^23,35,38,39^. Conversely, prior studies have shown that FuG-C and FuG-E efficiently transduce cells in the SNc^40,41^. Though pseudotyped lentivirus can transduce DA neurons in the nonhuman primate, certain serotypes produce an inflammatory response when combined with the ubiquitous MSCV promoter^33,42,43^. This study was designed to investigate the retrograde transduction efficiency and selectivity for dopaminergic cells of four different viral vectors, FuG-B2, FuG-C, FuG-E, and AAV2.retro.

## Materials and methods

### Vector preparation and storage

Lentivirus was produced at titers of 10^9^ I.U. using methods similar to those described previously^44^. 293 T (Lenti-X Invitrogen 632180, Life Technologies) cells were transfected with the Lenti-backbone plasmid and packaging plasmids (Addgene, Cambridge, MA, USA). The lentiviral vectors were pseudotyped with rabies virus glycoprotein variants (FuG-B2, FuG-C, and FuG-E) and engineered to express green or red fluorescent reporter proteins (GFP or RFP) under the human synapsin (hSyn) promoter, which allows for neuron-specific expression. AAV2.retro-hSyn1-GCAMP6f-P2A-nls-dTomato was provided by Jonathan Ting (Addgene plasmid # 51085; http://n2t.net/addgene:51085; RRID: Addgene_51085) and AAV2.retro-hSyn-CRE-eGFP was received from James M. Wilson (Addgene plasmid # 105540; http://n2t.net/addgene:105540; RRID: Addgene_105540) (Table 1).

### Subjects

All experimental procedures conformed to the Institute of Medicine Guide for the Care and Use of Laboratory Animals and were carried out under an Animal Study Proposal approved by the Animal Care and Use Committee of the National Institute of Mental Health. Subjects were 8 adult rhesus monkeys (*Macaca mulatta*: Monkey A, Monkey B, Monkey C, Monkey D, Monkey E, Monkey F, Monkey G, and Monkey H). All NHPs (6 females and 2 males) were between 4 and 15 years old, and their weights ranged from 5.3 to 11.4 kg (Table 1). They were housed on a 12-hour light-dark cycle with access to water ad libitum and fed a diet of monkey biscuits twice daily, approved treats, and fruit.

**Table 1:** Summary table of surgical cases. Virus was targeted to striatum in stereotaxic surgeries. Incubation is defined as the time between viral injection and post-mortem fixation. Expression as a percentage in the rhesus macaque brain at the site of local injection averaged across 3 anterior-posterior levels. Asterisk (*) indicates use of convection enhanced delivery.

NHP	Age	Sex	Weight (kg)	Construct	Hemisphere	Titer	Injections x volume (µL)	Incubation (weeks)	Striatal coverage (%)
A	10	F	6.3	FuG-B2-hSyn::mCherry FuG-C-hSyn::GFP	Right Left	2.00 × 10^9^ (iu/µL) 2.00 × 10^9^ (iu/µL)	12 × 10 µL 12 × 10 µL	6	7.80 6.20
B	11	F	11.4	FuG-B2-hSyn::GFP FuG-C-hSyn::mCherry	Left Right	1.00 × 10^9^ (iu/µL) 2.00 × 10^9^ (iu/µL)	12 × 10 µL 12 × 10 µL	6	1.90 6.90
C	13	F	5.6	FuGE-hSyn::mCherry	Right	2.00 × 10^9^ (iu/µL)	7 × 10 µL	8	4.00
D	15	F	8.8	FuGE-hSyn::GFP	Right	2.00 × 10^9^ (iu/µL)	7 × 10 µL	6	15.40
E	11	M	8.6	AAV2.retro-hSyn1-GCAMP6f-P2A-nls-dTomato	Left	1.00 × 10^13^ (vg/mL)	11 × 10 µL	62	1.12
F	10	F	6.5	AAV2.retro-hSyn1-GCAMP6f-P2A-nls-dTomato	Right	1.00 × 10^13^ (vg/mL)	5 × 10 µL	8	6.30
G	8	F	5.3	AAV2.retro-hSyn-CRE-eGFP	Bilateral	1.00 × 10^12^ (vg/mL)	1 × 80 µL*	6	N/A
H	4	M	7.0	AAV2.retro-hSyn-CRE-eGFP	Bilateral	1.00 × 10^12^ (vg/mL)	1 × 80 µL*	6	N/A

### Surgeries

**Fig. 1:**
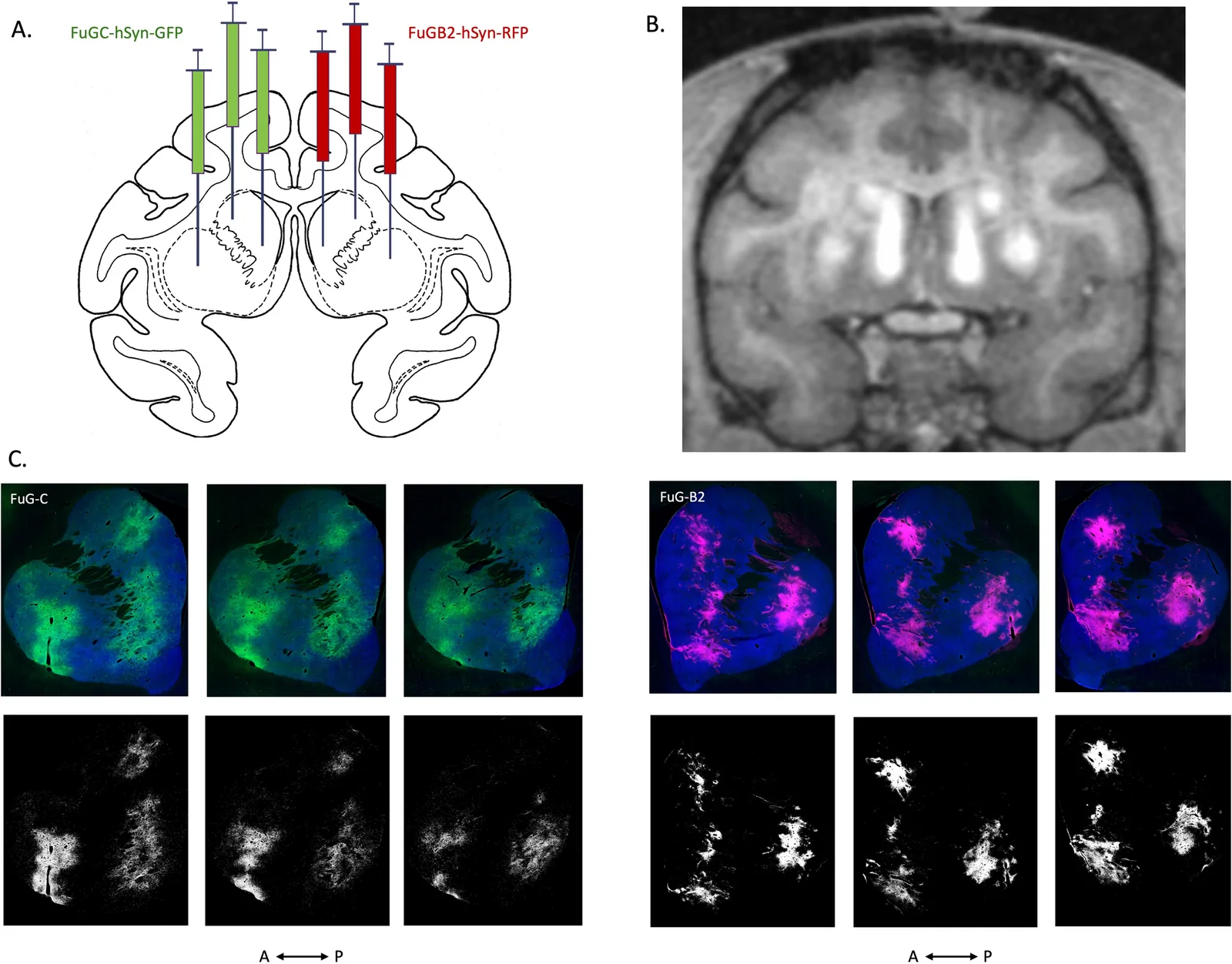
Viral delivery and expression in the striatum of a macaque brain. (**A**) Schematic illustrates striatal injection sites in Monkey A. FuG-C pseudotyped vector was delivered into the left hemisphere. FuG-B2 pseudotyped vector was delivered into the right hemisphere. (**B**) Same-day post-op MRI to visualize target accuracy via 0.2 mM manganese contrast agent co-infused with virus. (**C**) Reporter expression visualized post-mortem with immunohistochemistry and fluorescence microscopy across 3 consecutive anterior-posterior sections with 400 μm spacing. The image was thresholded using ‘intermodes’ on ImageJ (see methods) for quantification of the injection area (bottom row).

Surgeries were performed under aseptic conditions in a fully equipped operating suite under veterinary supervision. Prior to surgery, animals were sedated with ketamine hydrochloride (10 mg/kg, intramuscular) and midazolam (0.1 mg/kg, intramuscular). The NHPs were then intubated. A surgical level of anesthesia was induced and maintained with isoflurane (1–4%) throughout the procedures. Vital signs (body temperature, heart rate, blood pressure, SpO2, and expired CO_2_) were monitored. NHPs received antibiotic (Cefazolin) and isotonic fluids intravenously throughout the surgery. The brain was accessed by retracting muscle, removing a bone flap, and reflecting the dura mater. Stereotaxic injection coordinates were derived from pre-operative structural MRIs^45,46^. Post-operative MRI using manganese (Mn^2+^) contrast agent allowed for rapid post-operative visualization of viral vector delivery sites. The procedure has been described in detail previously^47^. Postoperative visualization with Mn^2+^ suggested needle occlusion in NHP B during intrastriatal delivery of the FuG-B2 pseudotyped vector (see Fig. S1). Virus aliquots were stored at −80 °C and were allowed to thaw on ice prior to injection. Virus was taken up into 100 µL Hamilton syringes (30G, beveled 45°, step) via a Harvard Apparatus Pump 11 Elite Nanomite. For monkeys A – F, between 70 and 150 µL of virus (10 µL/site at a rate of 0.5–1.0 µL/min) was delivered unilaterally into striatum across three anterior-posterior levels (interaural + 24 mm to + 19 mm), 1.5–2.0 mm apart, of each NHP. Injections were matched across hemispheres in the FuG-B2 and FuG-C cases (Fig. 1; Table 1). Injections along the same track were placed 2–2.5 mm apart in the dorso-ventral plane. For monkeys G and H, single injections of AAV2.retro-hSyn-CRE-eGFP were delivered bilaterally into the ventral striatum, specifically targeting the nucleus accumbens (NAc). Using a progressive increase in infusion rate known as convection enhanced delivery (CED), 80 µL of virus was injected into each hemisphere at a rate of 0.5 µL/min to 3–3.5 µL/min over the course of 45 min. Methods for CED have been described previously in detail^48^. To prevent infection and minimize swelling, NHPs A through F received dexamethasone sodium phosphate (0.4 mg/kg) and Cefazolin antibiotic (15 mg/kg) one day prior to surgery and in the week following the surgical date. Analgesic ketoprofen (10–15 mg) was given immediately following the surgery and for two days after. NHPs G and H were post-operatively monitored for 5–7 days by veterinary staff and received Cefazolin, Hydromorphone, and Buprenorphine (antibiotic and pain management).

### Tissue preparation and histological processing

After a minimum 6-week incubation period, the NHPs were euthanized. NHPs were administered ketamine hydrochloride (10 mg/kg), and were then intravenously given a lethal dose of sodium pentobarbital (100 mg/kg). Following euthanasia, the NHPs were transcardially perfused with 1 L of saline and 3 L of 4% paraformaldehyde in 0.1 M phosphate-buffered saline. Brains were extracted and refrigerated in 10% formalin. The brains were cryoprotected in buffered formalin/glycerol and then coronally sectioned with a freezing microtome into 40 μm slices. Sections were collected in series of 10. Every tenth section was mounted on gelatin-coated slides, defatted, stained with thionin, and cover-slipped. Two series were stained with 3,3’-diaminobenzidine (DAB) for RFP and GFP, respectively. A series was processed for fluorescence immunohistochemistry (IHC) (with the exception of monkeys G and H, which were stained for DAB only). For triple-fluorescence detection, NHP tissue sections were washed in PBS (3 × 10 min), blocked with 20% goat serum, then washed again in PBS (3 × 10 min) before overnight incubation at room temperature with primary antibodies: anti-GFP (Abcam AB13970, chicken, 1:5000), anti-mCherry (Abcam Ab167453, rabbit, 1:5000), and anti-TH (Immunostar 22941, mouse, 1:1000) diluted in PBS. Following primary incubation, sections were washed in PBS (3 × 10 min), then incubated for 2 h at room temperature with secondary antibodies: goat anti-chicken 488, goat anti-rabbit 555, and goat anti-mouse 647 (all Invitrogen, 1:300 in PBS), keeping sections in dark conditions to prevent photobleaching. After final PBS washes (3 × 10 min), sections were mounted with DPX and coverslipped, resulting in triple-label fluorescence with GFP, mCherry, and TH signals. A subset of AAV2.retro striatal sections exhibited signs of degradation, including loss of tissue integrity, loss of specific fluorescent signal, and elevated background, likely attributable to suboptimal preservation or age-related deterioration. These sections were unsuitable for analysis, and no previously acquired images of these sections were available as an alternative.

### Imaging and cell quantification

**Fig. 2:**
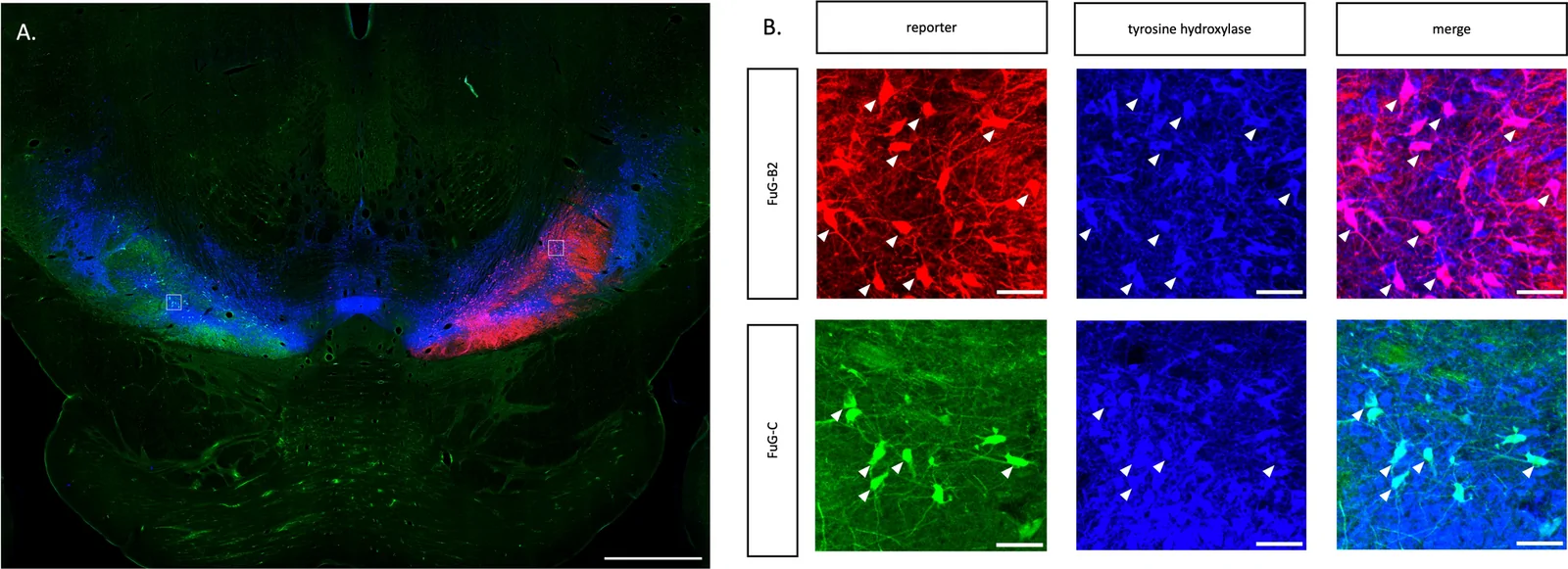
Co-expression of fluorescent reporter protein and TH antibody in the SN of a macaque visualized using immunohistochemistry and fluorescence microscopy. (**A**) Sample coronal section from Monkey A. Scale bar 2 mm. (**B**) High magnification images corresponding to the white boxes in A. Reporter and TH IHC in SN after transduction with FuG-B2 and FuG-C viral vectors. White arrows indicate individual retrogradely labeled cells co-expressing reporter protein and TH. Scale bar 100 μm.

**Fig. 3:**
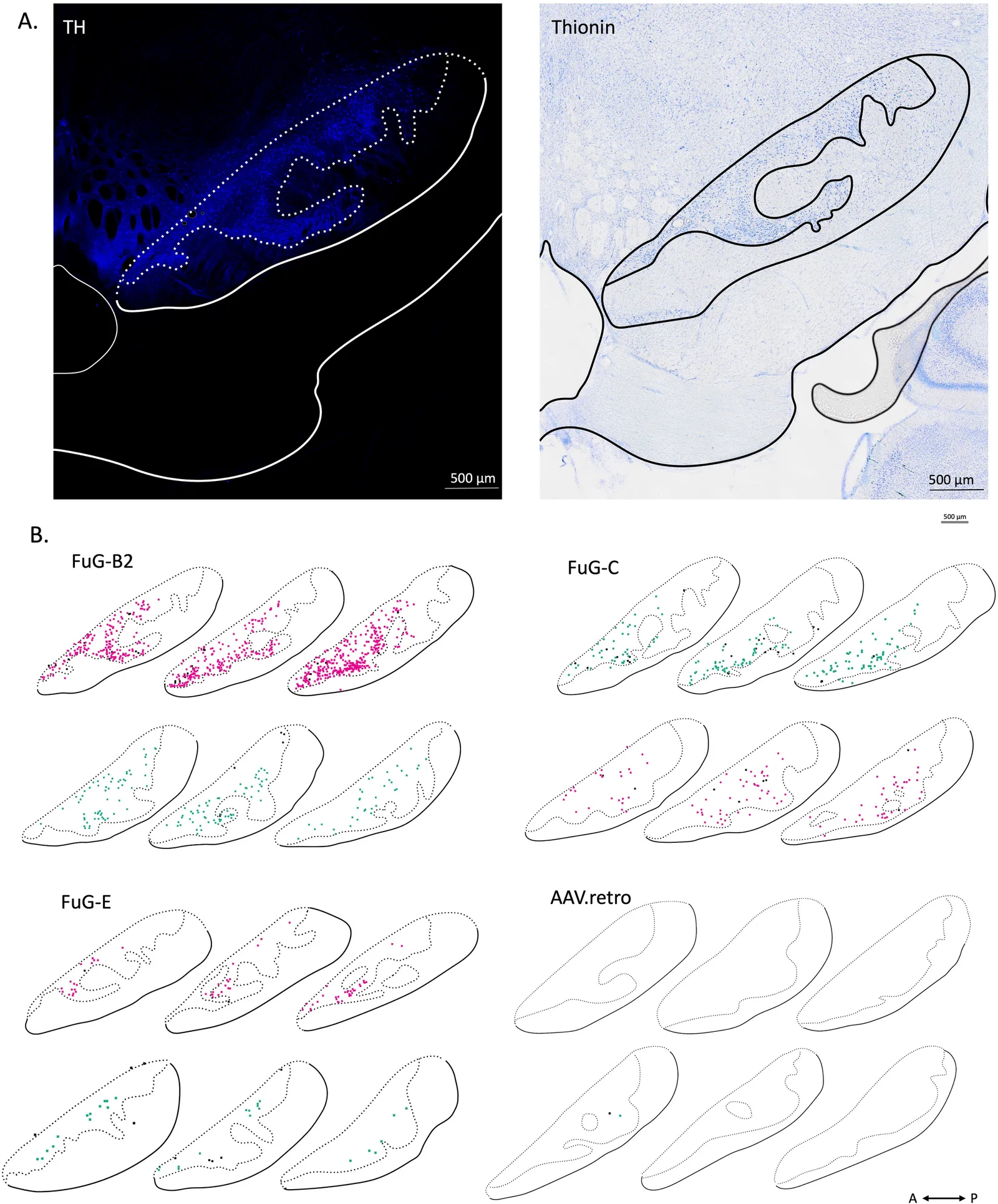
Retrograde expression within SNc. (**A**) SNc boundary (white dashed line) was determined by the distribution of TH-positive cells (left) and was validated by thionin IHC of a Sect. 160 μm rostral (right). Scale bar 500 μm. (**B**) Distribution of reporter-positive cells across three consecutive anterior-posterior levels. Cells co-expressing reporter and TH antibody are plotted in color (RFP or GFP), and cells expressing reporter but not TH antibody are plotted in black. Some sections are reflected across the vertical axis for visual symmetry (i.e. all sections shown as right hemisphere, including those acquired from the left hemisphere). Data collected from 6 monkeys: FuG-B2 - Monkey A (top), Monkey B (bottom); FuG-C - Monkey A (top), Monkey B (bottom); FuG-E - Monkey C (top), Monkey D (bottom); AAV2.retro - Monkey F (top), Monkey E (bottom). Scale bar 500 μm.

Mounted tissue sections were scanned with 10x (Figs. 1, 2 and 3, and Fig. S1) and 40x (validation) objectives using an Olympus VS200 scanning microscope with fluorescence microscopy. Fluor 488 and Fluor 555 antibodies were visualized under a 3 ms exposure, and Fluor 647 was scanned under a 40 ms exposure. Brightness, contrast, and exposure were held constant across cases. For previously acquired images, brightness and contrast were visually matched to newly scanned sections to ensure consistent presentation across cases. To minimize bias, cell identification criteria were established prior to quantification of any sections. Reporter-positive cells were counted independently before TH channel overlay, such that dopaminergic identity was confirmed only after initial quantification was complete. A conservative criterion was applied: only cells with labeling clearly distinguishable from background were counted. Ambiguous profiles, including dimly labeled cells and overlapping somata that could not be resolved individually, were excluded. The SNc boundary was determined using the cell distribution on a thionin-stained coronal section. The boundary was transposed onto a TH-stained fluorescent image of an adjacent series using anatomical landmarks and blood vessels. Boundaries were adjusted according to the dorsal/ventral distribution of TH-antibody positive cells. Using FIJI software^49^, total SNc area of the imaged tissue was quantified in mm^2^. Reporter expression in the SNc was quantified across three consecutive anterior-posterior sections within a series for each viral vector. Regions of high expression were selected to reflect the sparse striatal targeting; however, we acknowledge this may introduce a systematic bias resulting from the non-uniform cell distribution in the selected sections. Individual reporter-positive cells and dopaminergic cells were quantified and plotted using Adobe Illustrator. Images were separated channel-wise and later overlaid to validate reporter and TH-antibody co-expression per cell.

For quantification of expression at the injection site in striatum (interaural + 24 mm to + 19 mm), three consecutive coronal sections with the highest local expression were selected from each NHP and viral vector case. Exposure time, contrast, and brightness levels were uniformly matched across images. In FIJI, the striatum was outlined on a Fluor 647 section and quantified in square pixels. The injection area was visualized in the red or green channels (corresponding to the color of the reporter) using ‘Intermodes’ thresholding, since this approach is suited to images with bimodal intensity distribution, distinguishing labeled signal from background without requiring manual threshold adjustment, thereby improving reproducibility across sections. The injection area was quantified in square pixels as a percentage of the total striatal area. Due to the absence of fluorescent striatal sections in the AAV2.retro cases, injection site quantification was performed on DAB sections rather than fluorescent images. A similar procedure and thresholding technique was used in FIJI to evaluate local expression at the striatal injection site. Ratios between the quantified injection site area and the total area of the striatum were used to compare the levels of viral expression at the site of local injection between cases (Table 2). SN and VTA in the AAV2.retro-hSyn-CRE-eGFP cases (NHP G and H) were scanned using 20x extended depth of field (EDF) with a 10-micron Z-stack on a DAB-stained section (Fig. S2).

### Statistical analysis

The data was fitted in a hierarchical model under the Bayesian framework. The model was formulated as follows:

$${y_{m\left( t \right)s}} = {\mu _t} + {\theta _m} + {\varepsilon _{ms}}$$

$${\theta _{m~}}\sim N\left( {0,{\tau ^{\mathrm{2}}}} \right)$$

$${\varepsilon _{ms~}}\sim t\left( {0,{\sigma ^{\mathrm{2}}},\nu } \right)$$

The input data *y*_*m*(*t*)*s*_ denotes the cell counts per unit area on slice *s* (*s* = 1, 2, 3) of the *m*th monkey (*m* = 1, 2, …, 6) that belongs to *t*th treatment group (*t* = 1, 2, 3, 4). The parameters *µ*_*t*_, *θ*_*m*_, and *ε*_*ms*_ represent group-level effect, monkey-specific effect, and slice-level effect, respectively. Noninformative hyperprior was adopted for *µ*_*t*_, weakly-informative prior (i.e., Student’s half-*t*(3, 0, 1)) was used for standard deviations *τ* and *σ*. The hyperprior for the degrees of freedom, *v*, of the *t*-distribution for *ε*_*ms*_ was Γ(2,0.1).

The above hierarchical model was numerically solved using the R (R Core Team, 2024) package *brms*^50^. The resulting posterior samples were used to generate the six pairwise comparisons among the four treatment groups using the R package ggplot2^51^.

## Results

### Efficiency of viral vector transduction of SNc DA neurons

We injected four viral vectors unilaterally into the striatum of six NHPs (*n* = 2 hemispheres per vector) and compared their ability to efficiently transduce cells in the SN of the rhesus monkey (Fig. 1; Table 1). Post-operative visualization of the injected region revealed uniform signal (via Mn^2+^ contrast agent) in the target areas, consistent with planned trajectories (Fig. 1B), with the exception of the left hemisphere of NHP B (Fig. S1B-C). Post-mortem fluorescent IHC of reporter expression was consistent with post-operative Mn^2+^ signal distribution (Fig. 1C). Fluorescence microscopy confirmed successful retrograde transduction of neuronal populations following intrastriatal injection (Fig. 2A).

**Fig. 4:**
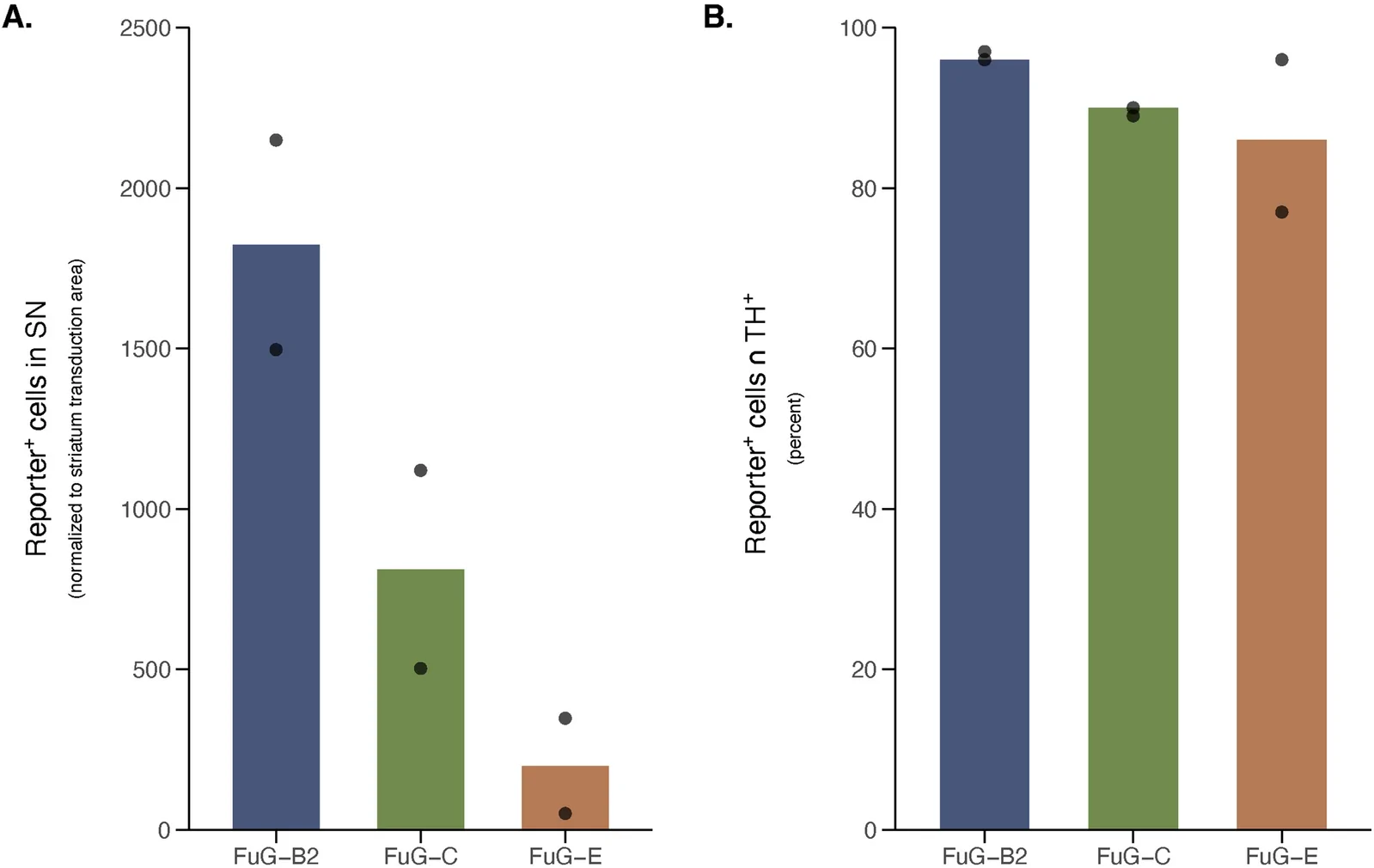
Viral vector expression and specificity. Data points show individual cases per treatment group. (**A**) Reporter-positive cells in SNc per unit of striatal area transduced (normalization is to cells per 1000 pixels of transduced striatal area). (**B**) Viral vector selectivity for TH-positive cells within SNc (i.e., of the reporter-positive cells, what percentage of the transduced cells were also TH-positive).

Analysis of individual channels and merged images revealed co-expression of reporter and TH-antibody, suggesting that the neuronal populations transduced were dopaminergic. Striatal expression was not predictive of which vectors would show significant transduction, as in some cases substantial intrastriatal coverage did not yield robust expression of reporter in DA cells (Table 2; Fig. 4).

**Fig. 5:**
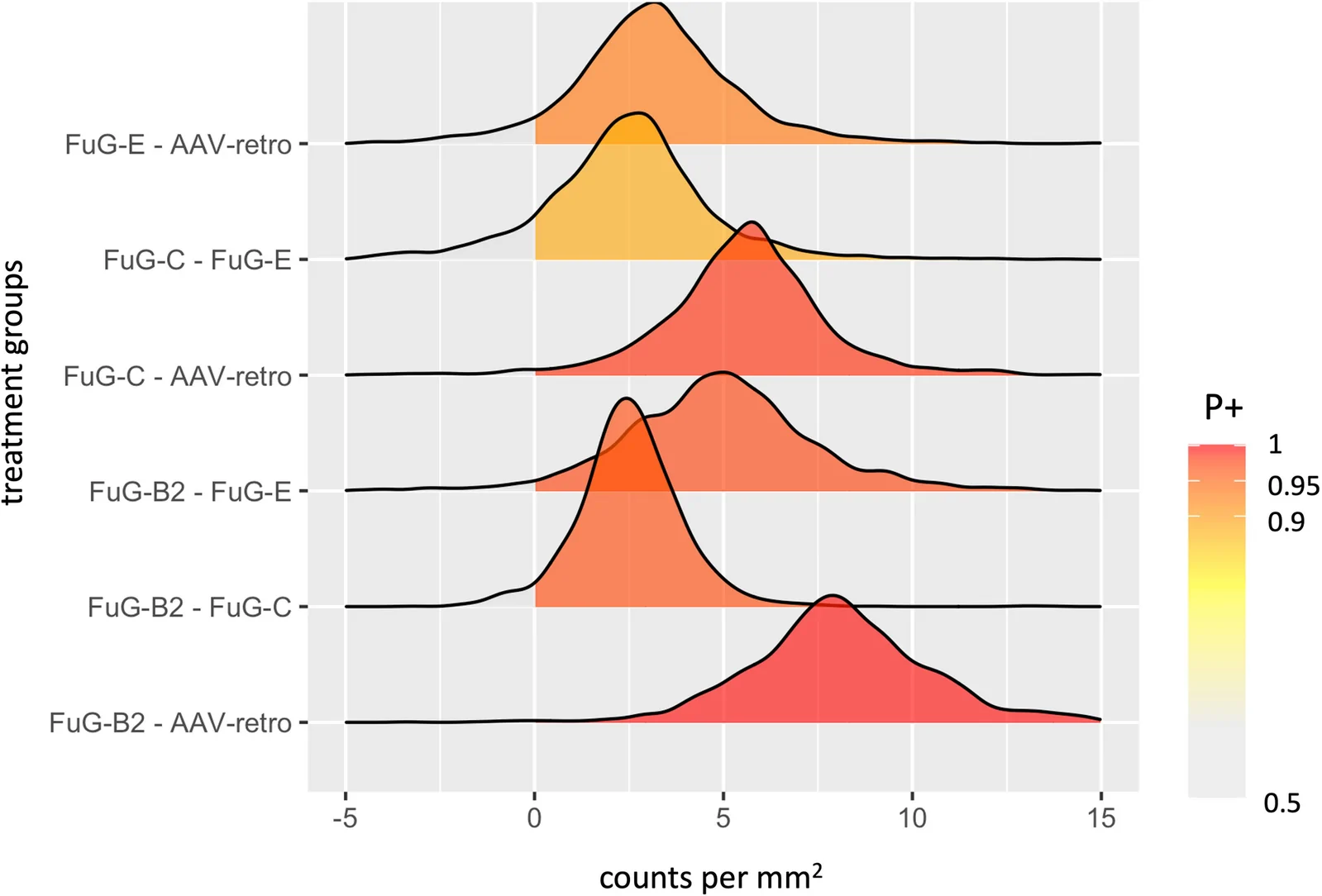
Bayesian modeling. Data was fitted in a hierarchical model under the Bayesian framework, representing different levels of positive effects between treatment groups. Input data indicates reporter^+^ cell counts per unit area on a slice (mm^2^) for each monkey assigned to a treatment group (*n* = 2 per treatment). The largest positive and consistent differences between treatment groups are indicated in red.

**Fig. 6:**
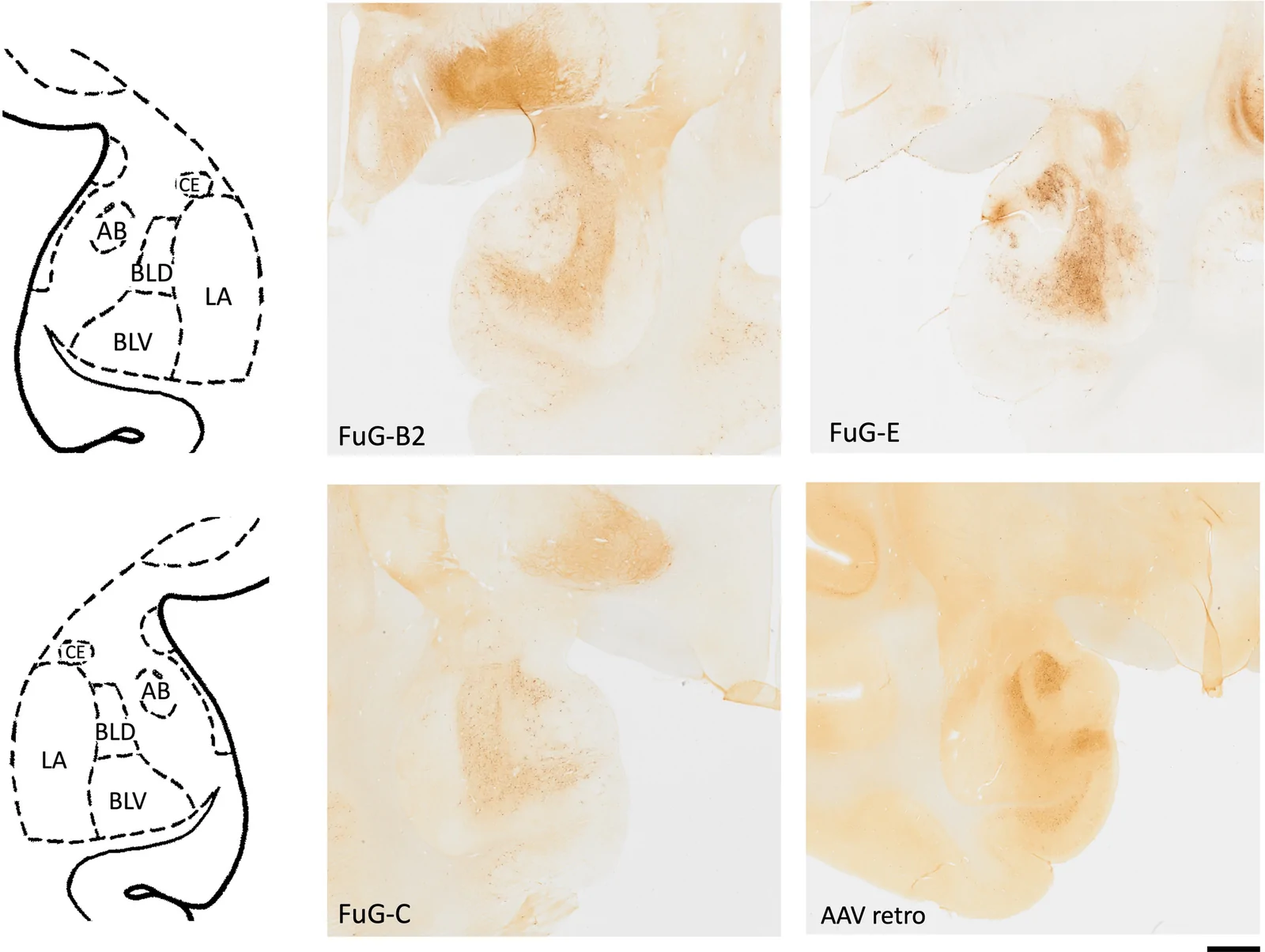
Transduced cells in the BLA following viral delivery into striatum. Schematic of amygdalar subdivisions (top – right hemisphere, bottom – left hemisphere). Retrograde expression within basolateral amygdala. Post-mortem DAB IHC visualized with brightfield microscopy. Scale bar 2 mm. AB – accessory basal nucleus; BLD – basolateral amygdala nucleus, dorsal; BLV – basolateral amygdala nucleus, ventral; LA – lateral amygdala; CE – central nucleus.

Reporter expression in the SNc was quantified across three consecutive anterior-posterior sections within a series for each viral vector (Fig. 3; Table 2). Relative retrograde transduction efficacy of dopaminergic cells was assessed by normalizing SNc cell counts to total striatal expression. The highest level of reporter expression in SNc was observed after intrastriatal delivery of FuG-B2 (‘HiRet’) (Fig. 4A; Tables 1 and 2). FuG-C and FuG-E produced comparable levels of transduction in SNc, though lower than FuG-B2 (Figs. 4A and 5; Table 2), despite similar striatal coverage (Table 1). AAV2.retro produced negligible expression in SNc (Figs. 3B and 4A; Table 2), despite expression in other brain regions, including the basolateral nucleus of the amygdala (BLA) (Fig. 6). The data was fitted to a hierarchical model under the Bayesian framework, accounting for variability between subjects and measuring the density of labeled cells in the tissue samples (Fig. 5). The pairwise comparisons reveal distinct performance hierarchies among the viral vectors. FuG-B2 produced significantly higher cell labeling density than AAV2.retro (difference = 166.2, posterior probability = 99.9%, *p* = 0.003). FuG-B2 also demonstrated superior performance to FuG-E with moderate consistency (difference = 147.7, posterior probability = 99.8%, *p* = 0.0008) and to FuG-C (difference = 177.7, posterior probability = 99.95%, *p* < 0.001). Taking into consideration both normalized cell counts in SN (Fig. 4A) and the model-estimated differences accounting for hierarchical structure and individual variability (Fig. 5), the retrograde transduction efficiency of the viral vectors can be ranked as follows: FuG-B2 > FuG-C > FuG-E > AAV2.retro. In our hands, convection enhanced delivery of the same capsid with a different transgene, AAV2.retro-hSyn-CRE-eGFP (NHPs G and H), to the NAc drove expression in the midbrain structure adjacent to SNc, the ventral tegmental area (VTA) (Fig. S2). The midbrain cells transduced in these two cases were not quantified or used for analysis, and dopaminergic identity would need to be validated with a stain against TH.

**Table 2:** Viral vector expression and selectivity in SNc. SNc area, cell counts, and percent co-expression are averaged across 3 anterior-posterior sections per monkey for each viral vector case.

Viral Vector	SNc Area (mm^2^)	Reporter^+^ Cells	Reporter^+^/TH^+^ Cells	Percent Co-expression
FuG-B2 (NHP A)	7.9	269	260	97
FuG-B2 (NHP B)	8.8	56	54	96
FuG-C (NHP A)	8.5	63	56	89
FuG-C (NHP B)	9.3	42	38	91
FuG-E (NHP C)	5.6	24	23	96
FuG-E (NHP D)	5.0	13	10	77
AAV.retro (NHP E)	7.1	2	1	50
AAV.retro (NHP F)	9.4	0	0	0

### Specificity of viral vector transduction in SNc

FuG-C and FuG-E pseudotypes demonstrate predominantly neuronal tropism, whereas FuG-B2 facilitates gene delivery to neurons and glial cells at the site of injection^32,33^. Fluorescent reporter sequences (RFP or GFP) in the transfer plasmid were expressed under the control of an hSyn promoter, which confers preferential transduction of neuronal cells, and thereby restricts expression to neurons in the FuG-B2 cases. FuG-B2 exhibited superior selectivity for TH-positive cells in the SNc (96.5%) as evidenced by co-localization of TH-antibody and reporter (Figs. 2B, 3B and 4B), suggesting an intrinsic tropism for dopaminergic neuronal populations. FuG-C also exhibited strong DA cell specificity (89.5%), but transduced other cell types, potentially GABAergic populations within SNc, at a higher rate than FuG-B2. FuG-E showed comparable DA-cell specificity (86.5%) to FuG-C (Fig. 4; Table 2).

### Expression and transduction outside of SNc

Although the pseudotyped FuG-B2 vector demonstrated superior efficiency and selectivity within the midbrain following intrastriatal injection, the other three vectors exhibited high transduction in different brain regions, such as cortex and basolateral amygdala (BLA). AAV2.retro and FuG-E demonstrated qualitatively comparable transduction levels of cells in the basolateral amygdala (BLA) to FuG-B2 and FuG-C (Fig. 6). This contrasts with the failure of AAV2.retro to transduce cells in the SNc, and with the weak FuG-E transduction in the midbrain. Two further NHPs (G and H) received convectionenhanced delivery of the same AAV2.retro capsid with a different transgene, AAV2.retro-hSyn-CRE-eGFP, into the NAc core and shell. Expression in SN and VTA was evaluated qualitatively (Fig. S2), and this virus appears to transduce cells in the midbrain effectively.

## Discussion

The present study investigated retrograde transduction efficiency and selectivity for dopaminergic cells in the SNc of the NHP using four different viral vectors, FuG-B2, FuG-C, FuG-E, and AAV2.retro. This study was exploratory in design, with titers selected based on lab protocols and prior literature rather than systematic dose-titration across vectors. Cross-vector comparisons of transduction efficiency are therefore interpreted with this limitation in mind. Combining a pan-neuronal promoter, human synapsin (hSyn), with FuG-B2 appeared to improve expression in neuronal populations relative to FuG-C and FuG-E.

### Viral vector safety

Viral infection of glial cells can trigger a pro-inflammatory response, with microglia (derived from myeloid precursors) and astrocytes playing central roles in neuroinflammation, potentially affecting their supportive functions in neuronal survival and increasing the risk of tumorigenesis^33,52,53^. Introducing an hSyn promoter to the construct reduces glial cell transduction at the site of local injection, reducing the risk of a pro-inflammatory response typically seen with transduction of non-neuronal cells^54,55^. Contrary to previous findings in which a ubiquitous promoter was used^33^, no gross changes in anatomical integrity were observed in this study following the delivery of the four test vectors, although more sensitive measures of inflammation and microglial changes would need to be quantified before using any of these vectors.

### AAV2.retro labeling inconsistencies in the midbrain

A recent study^23^ demonstrated successful retrograde transduction of DA populations in the NHP SNc following intrastriatal delivery of AAV2.retro, which failed to transduce DA cells in our hands. There are several factors which may have explained the discrepancy between Yan et al.’s finding and ours, including the use of a 2 A linker^56^ and a nuclear localization signal (NLS)^57^ in the present study. However, a previous study^35^ used AAV2.retro without an NLS or a 2 A linker and found dense terminal field labeling, but not labeling of cells in the SNc, ruling out these factors as the explanatory variable for the discrepancies between Yan et al.’s study and ours. However, factors beyond capsid-promoter interactions^58^(titer, brain area, injection method, species) may also dictate transduction patterns in the NHP, emphasizing the importance of construct composition and methods for replication of findings.

Convection enhanced delivery of the same AAV2.retro capsid with a different transgene, AAV2.retro-hSyn-CRE-eGFP (Fig. S2), successfully drove expression in the midbrain, specifically in the VTA in our hands. The transduction of these cell populations may be attributed to the specific targeting of the NAc shell and core, as well as construct composition (NHPs G and H). The identity of the transduced cells needs to be validated with more extensive staining. Targeting injections to the putamen and caudate with AAV2.retro (NHPs E and F) yielded sparse labeling in the SNc, consistent with Weiss et al. (2020). Weiss et al. used convection enhanced delivery in the striatum with AAV2.retro, suggesting that the injection method cannot be the attributing factor for successful expression in SNc. Our observation of AAV2.retro (NHPs E and F) expression in amygdala (Fig. S1) and cortex provides evidence that the striatal injections were successful in driving retrograde transduction, but the specific construct AAV2retro-hSyn1-GCAMP6f-P2A-nls-dTomato failed to express in DA cell populations of the SNc. New engineered AAV2 variants^59^ show promise for successful expression in NHP brain regions not consistently transduced by AAV2.retro, and may prove to be useful alternatives to the FuG-B2 vector pending more extensive evaluation. Consistency of results across macaque species should also be explored further. More broadly, differences in expression, axonal transport, and localization may be independently influenced by promoter type, reporter gene, and regulatory elements, and cannot be fully disentangled from capsid-specific effects in the present study. Additionally, variable post-injection survival periods across cases, including one AAV2.retro case with an extended incubation of 62 weeks, should be noted as a potential confound, though expression in this case was similarly sparse to the paired case with a more comparable survival period.

### Circuit and cell-type specificity

The utility of viral vector systems lies in their potential to deliver genetic tools with cell-type and circuit specificity. Leveraging retrograde transport to achieve efficient labeling of specific neuronal populations enables therapeutic interventions in primate models of neurodegenerative diseases^15^. Combining retrograde transport with cell-type specific expression allows for precise targeting of neuromodulatory inputs, thus providing a means of selectively altering circuit-level processing. Populations of neurons in the SNc that project to caudate and putamen are largely segregated^23,60^. These subpopulations may display differential vulnerability to pathology or play distinct roles within the circuit. Retrograde transduction with cell-type-selective enhancers, such as AiP13863^61,^ may be an effective means of targeting subpopulations of neurons in the SNc, ideal for microcircuit dissection at the level of basic research and for a ‘precision medicine’ approach in gene therapy. Our results suggest that FuG-B2 may be an optimal starting point; combining this vector with cell-type-selective enhancers has the potential to provide the selectivity required to exploit molecular genetic tools to their full potential for the study of the dopaminergic system.

## Supplementary Information

Below is the link to the electronic supplementary material.


Supplementary Material 1


## Data Availability

The raw data reported in the main text are available online through Figshare (https://doi.org/10.6084/m9.figshare.30898529).

## References

[1] Chen, H. et al. Dopaminergic system and neurons: Role in multiple neurological diseases. *Neuropharmacology* **260**, 110133 (2024). 39197818 10.1016/j.neuropharm.2024.110133

[2] Volkow, N. D., Fowler, J. S., Wang, G. J., Baler, R. & Telang, F. Imaging dopamine’s role in drug abuse and addiction. *Neuropharmacology* **56**, 3–8 (2009). 18617195 10.1016/j.neuropharm.2008.05.022 PMC2696819

[3] Damier, P., Hirsch, E. C., Agid, Y. & Graybiel, A. M. The substantia nigra of the human brain. II. Patterns of loss of dopamine-containing neurons in Parkinson’s disease. *Brain* **122**(Pt 8), 1437–1448 (1999). 10430830 10.1093/brain/122.8.1437

[4] Fearnley, J. M. & Lees, A. J. Ageing and Parkinson’s disease: Substantia nigra regional selectivity. *Brain* **114**(Pt 5), 2283–2301 (1991). 1933245 10.1093/brain/114.5.2283

[5] German, D. C., Manaye, K. F., Sonsalla, P. K. & Brooks, B. A. Midbrain dopaminergic cell loss in Parkinson’s disease and MPTP-induced parkinsonism: sparing of calbindin‐D_25k_ —containing cells^a^. *Ann. N. Y. Acad. Sci.* **648**, 42–62 (1992). 1353337 10.1111/j.1749-6632.1992.tb24523.x

[6] Karimi, M. & Perlmutter, J. S. The role of dopamine and dopaminergic pathways in dystonia: Insights from neuroimaging. *Tremor Other Hyperkinet Mov (N Y)* **5**, 280 (2015). 25713747 10.7916/D8J101XV PMC4314610

[7] Ribot, B. et al. Dystonia and dopamine: From phenomenology to pathophysiology. *Prog. Neurobiol.* **182**, 101678 (2019). 31404592 10.1016/j.pneurobio.2019.101678

[8] Björklund, A. & Dunnett, S. B. Dopamine neuron systems in the brain: An update. *Trends Neurosci.* **30**, 194–202 (2007). 17408759 10.1016/j.tins.2007.03.006

[9] Joel, D. & Weiner, I. The connections of the dopaminergic system with the striatum in rats and primates: An analysis with respect to the functional and compartmental organization of the striatum. *Neuroscience* **96**, 451–474 (2000). 10717427 10.1016/s0306-4522(99)00575-8

[10] Kelly, E. A., Contreras, J., Duan, A., Vassell, R. & Fudge, J. L. Unbiased stereological estimates of dopaminergic and GABAergic neurons in the A10, A9, and A8 subregions in the young male Macaque. *Neuroscience* **496**, 152–164 (2022). 35738547 10.1016/j.neuroscience.2022.06.018 PMC9329254

[11] Freeze, B. S., Kravitz, A. V., Hammack, N., Berke, J. D. & Kreitzer, A. C. Control of basal ganglia output by direct and indirect pathway projection neurons. *J. Neurosci.* **33**, 18531–18539 (2013). 24259575 10.1523/JNEUROSCI.1278-13.2013 PMC3834057

[12] Howe, M. W. & Dombeck, D. A. Rapid signalling in distinct dopaminergic axons during locomotion and reward. *Nature* **535**, 505–510 (2016). 27398617 10.1038/nature18942 PMC4970879

[13] Bromberg-Martin, E. S., Matsumoto, M. & Hikosaka, O. Dopamine in motivational control: Rewarding, aversive, and alerting. *Neuron* **68**, 815–834 (2010). 21144997 10.1016/j.neuron.2010.11.022 PMC3032992

[14] Haber, S. N. & Knutson, B. The reward circuit: Linking primate anatomy and human imaging. *Neuropsychopharmacol* **35**, 4–26 (2010). 10.1038/npp.2009.129 PMC3055449 19812543

[15] Chen, Y. et al. Circuit-specific gene therapy reverses core symptoms in a primate Parkinson’s disease model. *Cell* **186**, 5394–5410.e18 (2023). 37922901 10.1016/j.cell.2023.10.004

[16] El-Shamayleh, Y., Ni, A. M. & Horwitz, G. D. Strategies for targeting primate neural circuits with viral vectors. *J. Neurophysiol.* **116**, 122–134 (2016). 27052579 10.1152/jn.00087.2016 PMC4961743

[17] Gray, S. J., Woodard, K. T. & Samulski, R. J. Viral vectors and delivery strategies for CNS gene therapy. *Ther. Deliv.* **1**, 517–534 (2010). 22833965 10.4155/tde.10.50 PMC4509525

[18] Lerchner, W., Corgiat, B., Der Minassian, V., Saunders, R. C. & Richmond, B. J. Injection parameters and virus dependent choice of promoters to improve neuron targeting in the nonhuman primate brain. *Gene Ther.* **21**, 233–241 (2014). 24401836 10.1038/gt.2013.75 PMC6563815

[19] Perez, P., Chavret-Reculon, E., Ravassard, P. & Bouret, S. Using inhibitory DREADDs to silence LC neurons in monkeys. *Brain Sci.* **12**, 206 (2022). 35203969 10.3390/brainsci12020206 PMC8869890

[20] Isa, T. Double viral vector intersectional approaches for pathway-selective manipulation of motor functions and compensatory mechanisms. *Exp. Neurol.* **349**, 113959 (2022). 34953894 10.1016/j.expneurol.2021.113959

[21] Koshimizu, Y., Isa, K., Kobayashi, K. & Isa, T. Double viral vector technology for selective manipulation of neural pathways with higher level of efficiency and safety. *Gene Ther.* **28**, 339–350 (2021). 33432122 10.1038/s41434-020-00212-y PMC8221994

[22] Vancraeyenest, P. et al. Selective mesoaccumbal pathway inactivation affects motivation but not reinforcement-based learning in macaques. *Neuron* **108**, 568–581.e6 (2020). 32758424 10.1016/j.neuron.2020.07.013

[23] Yan, G. et al. Fluorescence detection of dopamine signaling to the primate striatum in relation to stimulus–reward associations. *Proc. Natl. Acad. Sci. U.S.A.* **122**, e2426861122 (2025). 10.1073/pnas.2426861122 PMC11929443 40080638

[24] Blömer, U. et al. Highly efficient and sustained gene transfer in adult neurons with a lentivirus vector. *J. Virol.* **71**, 6641–6649 (1997). 9261386 10.1128/jvi.71.9.6641-6649.1997 PMC191942

[25] Cockrell, A. S. & Kafri, T. Gene delivery by lentivirus vectors. *Mol. Biotechnol.* **36**, 184–204 (2007). 17873406 10.1007/s12033-007-0010-8

[26] Naldini, L., Blömer, U., Gage, F. H., Trono, D. & Verma, I. M. Efficient transfer, integration, and sustained long-term expression of the transgene in adult rat brains injected with a lentiviral vector. *Proc. Natl. Acad. Sci.* **93**, 11382–11388 (1996). 8876144 10.1073/pnas.93.21.11382 PMC38066

[27] Chan, K. Y. et al. Engineered AAVs for efficient noninvasive gene delivery to the central and peripheral nervous systems. *Nat. Neurosci.* **20**, 1172–1179 (2017). 28671695 10.1038/nn.4593 PMC5529245

[28] *Vectorology for Optogenetics and Chemogenetics* Vol. 195 (Springer US, 2023).

[29] Tervo, D. G. R. et al. A designer AAV variant permits efficient retrograde access to projection neurons. *Neuron* **92**, 372–382 (2016). 27720486 10.1016/j.neuron.2016.09.021 PMC5872824

[30] Carpentier, D. C. J. et al. Enhanced pseudotyping efficiency of HIV-1 lentiviral vectors by a Rabies/Vesicular Stomatitis Virus chimeric envelope glycoprotein. *Gene Ther.* **19**, 761–774 (2012). 21900965 10.1038/gt.2011.124

[31] Cronin, J., Zhang, X. Y. & Reiser, J. Altering the tropism of lentiviral vectors through pseudotyping. *CGT* **5**, 387–398 (2005). 10.2174/1566523054546224 PMC1368960 16101513

[32] Kato, S. & Kobayashi, K. Pseudotyped lentiviral vectors for tract-targeting and application for the functional control of selective neural circuits. *J. Neurosci. Methods* **344**, 108854 (2020). 32663549 10.1016/j.jneumeth.2020.108854

[33] Tanabe, S. et al. A note on retrograde gene transfer efficiency and inflammatory response of lentiviral vectors pseudotyped with FuG-E vs. FuG-B2 glycoproteins. *Sci. Rep.* **9**, 3567 (2019). 30837514 10.1038/s41598-019-39535-1 PMC6400974

[34] Chen, S.-H., He, B., Singh, S. & Martin, N. P. Vector Tropism. In *Vectorology for Optogenetics and Chemogenetics* (eds Eldridge, M. A. G. & Galvan, A.) 105–123. 10.1007/978-1-0716-2918-5_6. (Springer US, 2023).

[35] Cushnie, A. K. et al. Using rAAV2-retro in rhesus macaques: Promise and caveats for circuit manipulation. *J. Neurosci. Methods* **345**, 108859 (2020). 32668316 10.1016/j.jneumeth.2020.108859 PMC7539563

[36] Liu, Q., Wu, Y., Wang, H., Jia, F. & Xu, F. Viral tools for neural circuit tracing. *Neurosci. Bull.* **38**, 1508–1518 (2022). 36136267 10.1007/s12264-022-00949-z PMC9723069

[37] Wang, J. & Zhang, L. Retrograde axonal transport property of adeno-associated virus and its possible application in future. *Microbes Infect.* **23**, 104829 (2021). 33878458 10.1016/j.micinf.2021.104829

[38] Albaugh, D. L., Smith, Y. & Galvan, A. Comparative analyses of transgene expression patterns after intra-striatal injections of rAAV2-retro in rats and rhesus monkeys: A light and electron microscopic study. *Eur. J. Neurosci.* **52**, 4824–4839 (2020). 33113247 10.1111/ejn.15027 PMC7902345

[39] Weiss, A. R., Liguore, W. A., Domire, J. S., Button, D. & McBride, J. L. Intra-striatal AAV2.retro administration leads to extensive retrograde transport in the rhesus macaque brain: Implications for disease modeling and therapeutic development. *Sci. Rep.* **10**, 6970 (2020). 32332773 10.1038/s41598-020-63559-7 PMC7181773

[40] Kato, S., Kobayashi, K. & Kobayashi, K. Improved transduction efficiency of a lentiviral vector for neuron-specific retrograde gene transfer by optimizing the junction of fusion envelope glycoprotein. *J. Neurosci. Methods*. **227**, 151–158 (2014). 24613797 10.1016/j.jneumeth.2014.02.015

[41] Kato, S. et al. Selective neural pathway targeting reveals key roles of thalamostriatal projection in the control of visual discrimination. *J. Neurosci.* **31**, 17169–17179 (2011). 22114284 10.1523/JNEUROSCI.4005-11.2011 PMC6623855

[42] Kato, S. et al. A lentiviral strategy for highly efficient retrograde gene transfer by pseudotyping with fusion envelope glycoprotein. *Hum. Gene Ther.* **22**, 197–206 (2011). 20954846 10.1089/hum.2009.179

[43] Tanabe, S. et al. The use of an optimized chimeric envelope glycoprotein enhances the efficiency of retrograde gene transfer of a pseudotyped lentiviral vector in the primate brain. *Neurosci. Res.* **120**, 45–52 (2017). 28257798 10.1016/j.neures.2017.02.007

[44] Lerchner, W., Luz-Ricca, A., Dash, K., DerMinassian, V. & Richmond, B. J. Production, Testing, and Verification of Lentivirus for Regional Targeting in the Old-World Monkey Brain. In *Vectorology for Optogenetics and Chemogenetics* (eds Eldridge, M. A. G. & Galvan, A.) 3–15. 10.1007/978-1-0716-2918-5_1 (Springer US, 2023).

[45] Saunders, R. C., Aigner, T. G. & Frank, J. A. Magnetic resonance imaging of the rhesus monkey brain: use for stereotactic neurosurgery. *Exp. Brain Res.* **81**, (1990). 10.1007/BF00228139 2204546

[46] Walbridge, S., Murad, G. J. A., Heiss, J. D., Oldfield, E. H. & Lonser, R. R. Technique for enhanced accuracy and reliability in non-human primate stereotaxy. *J. Neurosci. Methods*. **156**, 310–313 (2006). 16516975 10.1016/j.jneumeth.2006.01.025 PMC4294192

[47] Fredericks, J. M. et al. Methods for mechanical delivery of viral vectors into rhesus monkey brain. *J. Neurosci. Methods.* **339**, 108730 (2020). 32302596 10.1016/j.jneumeth.2020.108730 PMC7238764

[48] Lee, S. et al. Synthetic serum markers enable noninvasive monitoring of gene expression in primate brains. 06.01.657212 Preprint at 10.1101/2025.06.01.657212 (2025). PMC13048365 41763204

[49] Schindelin, J. et al. Fiji - an Open Source platform for biological image analysis. *Nat. Methods*. **9** 10.1038/nmeth.2019 (2012). PMC3855844 22743772

[50] Bürkner, P. C. brms: An *R* package for bayesian multilevel models using *Stan*. *J. Stat. Soft* **80**, (2017).

[51] Wickham, H. *Ggplot2*. 10.1007/978-3-319-24277-4 (Springer International Publishing, 2016).

[52] Kobayashi, K. et al. Pseudotyped lentiviral vectors for retrograde gene delivery into target brain regions. *Front. Neuroanat.* **11**, 65 (2017). 10.3389/fnana.2017.00065 PMC5539090 28824385

[53] Rosario, A. M. et al. Microglia-specific targeting by novel capsid-modified AAV6 vectors. *Mol. Ther.* **3**, 16026 (2016). 10.1038/mtm.2016.26 PMC4909093 27308302

[54] Ciesielska, A. et al. Cerebral infusion of AAV9 vector-encoding non-self proteins can elicit cell-mediated immune responses. *Mol. Ther.* **21**, 158–166 (2013). 22929660 10.1038/mt.2012.167 PMC3538301

[55] Samaranch, L. et al. AAV9-mediated expression of a non-self protein in nonhuman primate central nervous system triggers widespread neuroinflammation driven by antigen-presenting cell transduction. *Mol. Ther.* **22**, 329–337 (2014). 24419081 10.1038/mt.2013.266 PMC3918916

[56] Lewis, J. E. et al. The use of a viral 2A sequence for the simultaneous over-expression of both the *vgf* gene and enhanced green fluorescent protein (eGFP) *in vitro* and *in vivo*. *J. Neurosci. Methods.* **256**, 22–29 (2015). 26300182 10.1016/j.jneumeth.2015.08.013 PMC4659456

[57] Earley, L. F. et al. Identification and characterization of nuclear and nucleolar localization signals in the adeno-associated virus serotype 2 assembly-activating protein. *J. Virol.* **89**, 3038–3048 (2014). 25552709 10.1128/JVI.03125-14 PMC4337552

[58] Bohlen, M. O. et al. Adeno-associated virus capsid-promoter interactions in the brain translate from rat to the nonhuman primate. *Hum. Gene Ther.* **31**, 1155–1168 (2020). 32940068 10.1089/hum.2020.196 PMC7698875

[59] Dzhashiashvili, Y. et al. Administration of barcoded AAV capsid library to the putamen of non-human primates identifies variants with efficient retrograde transport. 07.07.663544 Preprint at 10.1101/2025.07.07.663544 (2025). PMC12974201 41376158

[60] Haber, S. N., Fudge, J. L. & McFarland, N. R. Striatonigrostriatal pathways in primates form an ascending spiral from the shell to the dorsolateral striatum. *J. Neurosci.* **20**, 2369–2382 (2000). 10704511 10.1523/JNEUROSCI.20-06-02369.2000 PMC6772499

[61] Hunker, A. C. et al. Enhancer AAV toolbox for accessing and perturbing striatal cell types and circuits. *Neuron* **113**, 1507–1524e17 (2025). 40403704 10.1016/j.neuron.2025.04.035 PMC12237622

