# Supplementary material for: Retrograde transduction of dopaminergic cells in substantia nigra of the rhesus monkey
Source: Sci Rep. 2026 Jun 16;16:27492. doi: 10.1038/s41598-026-55097-5 (PMC13534416; doi:10.1038/s41598-026-55097-5)
Supplement: Supplementary file 1 [file 41598_2026_55097_MOESM1_ESM.docx]

**Supplementary Figures**

**Supplemental Figure 1**. Viral delivery and expression in the striatum of a macaque brain. (A) Schematic illustrates striatal injection sites in Monkey B. FuG-B2 was delivered into the left hemisphere and FuG-C was delivered into the right hemisphere (counter-balanced with Monkey A). (B) Same-day post-op MRI to visualize target accuracy via 0.2 mM manganese contrast agent co-infused with virus. (C) Reporter expression visualized post-mortem with immunohistochemistry and fluorescence microscopy across 3 consecutive anterior-posterior sections with 400 micron spacing. The striatum expression was thresholded using ImageJ for quantification of the injection area (bottom row).

**Supplemental Figure 2.** Viral expression in the VTA and SN of a macaque brain following convection enhanced delivery to the NAc (core and shell). 80 µl of AAV2.retro-hSyn-CRE-eGFP (titer 1 x 10^12^ ) was delivered bilaterally to the ventral striatum. Expression visualized 6 weeks post-mortem. Slice thickness 50 µm. Scale bar 500 µm.
